# Discrimination between possible sarcopenia and metabolic syndrome using the arterial pulse spectrum and machine-learning analysis

**DOI:** 10.1038/s41598-022-26074-5

**Published:** 2022-12-12

**Authors:** Li-Wei Wu, Te OuYoung, Yu-Chih Chiu, Ho-Feng Hsieh, Hsin Hsiu

**Affiliations:** 1 Division of Family Medicine, Department of Family and Community Medicine, Tri-Service General Hospital, School of Medicine, National Defense Medical Center, Taipei, Taiwan; 2 Health Management Center, Department of Family and Community Medicine, Tri-Service General Hospital, National Defense Medical Center, Taipei, Taiwan; 3 Graduate Institute of Biomedical Engineering, National Taiwan University of Science and Technology, No.43, Section 4, Keelung Road, Taipei, 10607 Taiwan; 4 Biomedical Engineering Research Center, National Defense Medical Center, Taipei, Taiwan

**Keywords:** Health care, Engineering

## Abstract

Sarcopenia is defined as decreased skeletal muscle mass and function, and is an important cause of frailty in the elderly, also being associated with vascular lesions and poor microcirculation. The present study aimed to combine noninvasive pulse measurements, frequency-domain analysis, and machine learning (ML) analysis (1) to determine the effects on the pulse waveform induced by sarcopenia and (2) to develop discriminating models for patients with possible sarcopenia. Radial blood pressure waveform (BPW) signals were measured noninvasively for 1 min in 133 subjects who visited Tri-Service General Hospital for geriatric health checkups. They were assigned to a robust group and a possible-sarcopenia group that combined dynapenia, presarcopenia, and sarcopenia. Two classification methods were used: ML analysis and a self-developed scoring system that used 40 harmonic pulse indices as features: amplitude proportions and their coefficients of variation, and phase angles and their standard deviations. Significant differences were found in several spectral indices of the BPW between possible-sarcopenia and robust subjects. Threefold cross-validation results indicated excellent discrimination performance, with AUC equaling 0.77 when using LDA and 0.83 when using our scoring system. The present noninvasive and easy-to-use measurement and analysis method for detecting sarcopenia-induced changes in the arterial pulse transmission condition could aid the discrimination of possible sarcopenia.

## Introduction

Sarcopenia is defined as decreased skeletal muscle mass and function, and is an important cause of frailty in the elderly^1–5^. It has been reported that around 50 million people worldwide have sarcopenia, which affects 13% of individuals aged 60–70 years^6,7^. Sarcopenia can affect whole-body metabolism, the inflammatory response, muscle force, functional capacity, and the amount of body fat^1^. Its progression is closely associated with old age, skeletal muscle disuse, malnutrition, chronic systemic inflammation, and anabolic disorder^8^. Aging is generally accompanied by changes in body composition (e.g., a loss of skeletal muscle mass), decreased physical activity and protein intake, and decreased concentrations of anabolic hormones^4,7^. Sarcopenia is associated with increased risks of falls and fractures in elderly individuals, which may further increase the mortality rates of elderly populations^9^. The consequences of sarcopenia also include increased physical disability, lower quality of life, higher mortality, and increased rates of cardiovascular disease (CVD) with a poor prognosis^2,6,7^.

Sarcopenia is believed to be associated with imbalances in the anabolic and catabolic pathways that regulate muscle protein synthesis^7^. Socioeconomic factors, physical activity, chronic diseases, and nutritional factors are risk factors for sarcopenia^9^. Sarcopenia is often related to multiple pathologies, such as type-2 diabetes mellitus, cancer, and obesity^4,7^. It also interacts bidirectionally with CVDs, such as by impairing exercise capacity in the elderly, which is strongly associated with CVDs, and CVDs and sarcopenia can coexist to further reduce exercise tolerance and increase mortality. Sarcopenia may lead to poor physical performance and reduced cardiorespiratory fitness in older patients with CVDs, while CVDs may correspondingly induce sarcopenia through common pathogenetic pathways to influence each other (e.g., hormonal changes, malnutrition, and physical inactivity)^8^.

Associations have been found between sarcopenia and various types of CVDs. Lower muscle strength is associated with increases in metabolic syndrome (MetS) and type-2 diabetes mellitus^1^. Sarcopenia has been noted to be significantly associated with poor outcomes, cardiovascular events, and impaired microcirculation in patients with type-2 diabetes mellitus^10^. Peripheral arterial disease (PAD) is also noted to be accompanied by musculoskeletal abnormalities including sarcopenia^11^. PAD and sarcopenia can run in parallel, perhaps since these conditions may act synergistically to decrease mobility. Males with PAD and sarcopenia demonstrated impaired PAD-specific mobility^6^.

The substantial impact of chronic musculoskeletal disorders on health make sarcopenia an important determinant of the health status^5^, and so an effective tool for accurate and timely diagnoses of sarcopenia is urgently needed. Several technologies are currently employed to estimate muscle mass, including bioimpedance analysis and anthropometry measures^4^. Imaging-based quantification using methods such as MRI and CT is considered the gold standard, but these methods have drawbacks of high cost, low accessibility, and radiation exposure^3,4^. Techniques measuring muscle function have also been used, including to evaluate muscle strength (e.g., grip strength and knee flexion strength) and the physical performance (e.g., gait speed, chair stand test, and walking speed)^4,7^.

Increased arterial stiffness is associated with the body composition in elderly males, such as a decreased skeletal muscle mass. It is possible that increases in the arterial stiffness can lower the perfusion efficiency, decrease the flows of blood, oxygen, and nutrients to muscle tissue, and hence lead to decreased muscle mass^2^. Since the transmission of an arterial pulse can be affected by the arterial stiffness, measuring and analyzing the pulse waveform can potentially aid the detection of abnormalities induced by sarcopenia and hence facilitate its early diagnosis and personalized follow-up. Previous noninvasive measurements revealed that the brachial-ankle pulse-wave velocity (PWV) increases as the skeletal muscle mass decreases^2^. Another study involving elderly Americans found that the carotid-femoral PWV was negatively correlated with skeletal muscle mass^8^.

The characteristics of the pulse waveform transmitting along an artery can reflect changes in the elastic properties of the arterial system, and this waveform can be easily measured noninvasively using wearable devices. It is therefore considered a useful method for monitoring physiological and pathological conditions, with many studies having investigated the detection of changes in arterial stiffness induced by various kinds of chronic diseases. For example, time-domain analysis has been used to study the effects induced in aged people and patients with hypertension, hypercholesterolemia, coronary artery disease, and PAD^12–16^. Frequency-domain analysis has also been used to investigate specific patterns in the pulse waveform induced by CVDs as well as noncardiovascular diseases such as cerebrovascular disease, MetS, dementia, vascular aging, and frozen shoulder^17–22^.

There is increasing interest in applying machine learning (ML) analysis to monitor health outcomes, since ML analysis can detect subtle changes in the pulse waveform. The present study aimed to combine noninvasive measurements, frequency-domain analysis, and ML analysis (1) to determine the effects on the pulse waveform induced by sarcopenia and (2) to verify the accuracy and validity of discriminating models developed for patients with sarcopenia. The present findings might be useful for developing a new noninvasive method for detecting sarcopenia-induced changes in the arterial pulse transmission condition and hence the vascular elastic properties. Furthermore, MetS is a cluster of pathological signs that not only increase the risk of the subsequent development of CVD, but might also be associated with sarcopenia. An association has been noted between changes in skeletal muscle mass and MetS development^1^. Data acquired from the recruited subjects were divided into subgroups to further understand how MetS might influence pulse-waveform analyses of sarcopenia.

## Materials and methods

**Figure 1:**
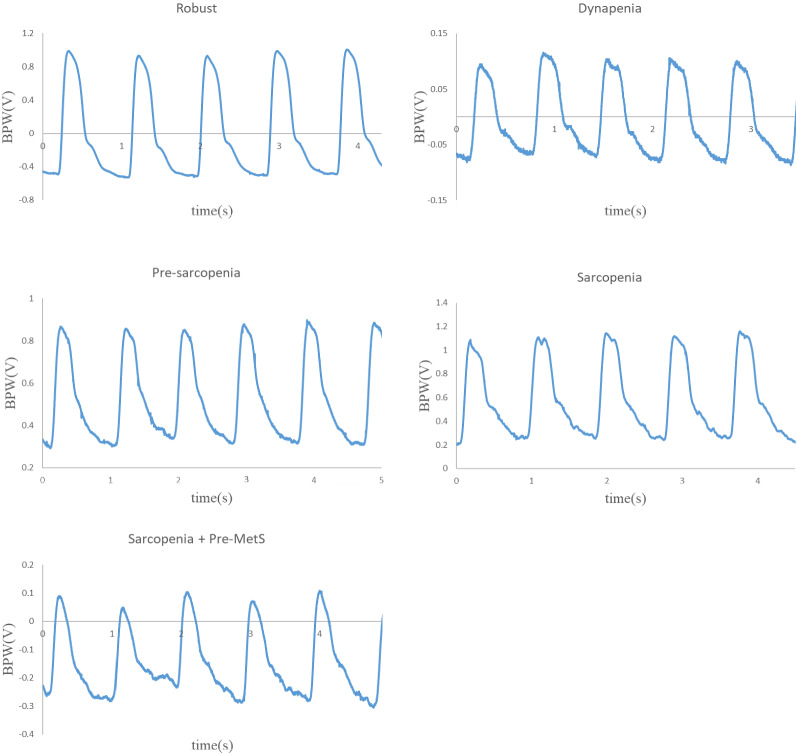
Typical BPW waveforms measured (in arbitrary units).

Details of the present experimental setup and the signal processing methods have been reported previously^21,22^ and are also available in the Supplemental Materials (Supplementary Information 1). Blood pressure waveform (BPW) and ECG signals were measured noninvasively (typical waveforms are shown in Fig. 1). HR_CV was calculated from the R−R intervals of the ECG signals as the coefficient of variation of the heart rate (HR) to evaluate changes in the HR variability. We applied frequency-domain analysis to derive 40 harmonic indices from the BPW signal based on the following 4 parameters for *n* = 1–10: amplitude proportion (*C*_*n*_), coefficient of variation of *C*_*n*_ (*CV*_*n*_), phase angle (*P*_*n*_), and standard deviation (SD) of *P*_*n*_ (*P*_*n*__*SD*).

Measurements were made in 133 volunteers whose characteristics are listed in Table 1. The study population was recruited at the Health Management Center of Tri-Service General Hospital from August 2020 to October 2021. The study was reviewed and approved by the institutional review board of Tri-Service General Hospital (TSGHIRB 2-108-05-161). All experiments were performed in accordance with relevant guidelines and regulations. Adults aged 71.01 ± 4.14 years (mean ± SD) who came for geriatric health checkups were eligible for inclusion in the study. The exclusion criteria were (1) severe illness such as liver cirrhosis, kidney failure, heart failure, cancer, or cerebral vascular disease, (2) severe mental illness, (3) severe endocrine disease such as hyper- or hypothyroidism, (4) skin ulceration or wound that could interfere the measurements, or (5) taking a medication that could affect vascular endothelial function. The eligible participants were invited by study assistants to enroll in this study voluntarily. All participants were informed about the study and then signed consent forms.

**Table 1:** Characteristics of the study subjects in the robust, dynapenia, presarcopenia, and sarcopenia groups.

Characteristics of the study participants	Groups					
	Robust	Dynapenia	Pre-sarcopenia	Sarcopenia	Total	*P* value
	*n* = 74	*n* = 28	*n* = 17	*n* = 14	*n* = 133	
**Continuous variables**						
Age (years), mean (SD)	70.20 (3.84)	72.71 (4.53)	69.65 (2.84)	73.57 (5.00)	71.01 (4.14)	0.208
BMI (kg/m^2^), mean (SD)	25.03 (2.12)	24.95 (2.19)	20.500 (1.64)	22.500 (2.01)	24.17 (2.43)	0.680
Abdominal circumference(cm) , mean (SD)	82.15 (6.45)	81.93 (6.37)	71.29 (5.91)	77.79 (6.50)	80.26 (6.83)	0.975
Systolic blood pressure (mmHg), mean (SD)	136.64 (14.13)	130.14 (13.65)	124.47 (7.86)	131.57 (12.57)	133.18 (13.29)	0.077
Diastolic blood pressure (mmHg), mean (SD)	80.26 (9.14)	75.79 (7.50)	73.94 (8.08)	76.36 (7.60)	78.10 (8.60)	0.650
Sleep (hour) , mean (SD)	6.76 (0.89)	6.45 (0.95)	6.76 (0.84)	6.50 (0.79)	6.67 (0.89)	0.841
Pulse (/min) , mean (SD)	71.86 (7.14)	73.82 (8.15)	76.64 (9.00)	70.71 (5.90)	73.18 (8.02)	0.325
**Clinical indicators**						
Gender (male), *n* (%)	38 (51.35)	10 (35.71)	4 (23.53)	2 (14.29)	54 (40.60)	0.019
Hypertension, *n* (%)	11 (14.86)	5 (17.86)	2 (11.76)	1 (7.14)	19 (14.29)	0.805
Type 2 diabetes mellitus, *n* (%)	11 (10)	5 (10.64)	3 (12.5)	1 (5)	20 (9.95)	0.976
Malignancy, *n* (%)	5 (6.76)	1 (3.57)	2 (11.76)	3 (21.43)	11 (8.27)	0.212
Coronary heart disease, *n* (%)	4 (3.64)	2 (4.26)	1 (4.17)	1 (5)	8 (3.98)	0.941
Chronic obstructive pulmonary disease, *n* (%)	1 (1.35)	1 (3.57)	1 (5.88)	2 (14.29)	5 (3.76)	0.128
Smoking, *n* (%)	16 (21.62)	3 (10.71)	3 (17.65)	2 (14.29)	24 (18.05)	0.616
Osteoporosis, *n* (%)	5 (4.55)	7 (14.89)	3 (12.50)	0 (0)	15 (7.46)	0.377

Differences were tested using one-way ANOVA.

According to the criteria listed in Table 2, the subjects were categorized into robust, dynapenia, presarcopenia, and sarcopenia groups. Furthermore, to investigate the possible effects of MetS in sarcopenia subjects, the subjects were assigned to a pre-MetS group (with one or two MetS factors) and a no-MetS group (with no MetS factor) according to the following criteria of MetS: (1) waist circumference ≥ 90 cm for males and ≥ 80 cm for females; (2) blood pressure (BP) ≥ 130/85 mmHg, or taking physician-prescribed hypertension treatment drugs; (3) low high-density lipoprotein cholesterol < 40 mg/dL for men and < 50 mg/dL for women; (4) fasting blood sugar value ≥ 100 mg/dL, or taking medicine prescribed by a doctor to treat diabetes; (5) high fasting triglyceride ≧ 150 mg/dL, or taking a physician-prescribed triglyceride-lowering drug.

**Table 2:** Criteria for inclusion in the robust, dynapenia, presarcopenia, and sarcopenia groups.

Robust	Dynapenia	Pre-sarcopenia	Sarcopenia	
×	✓	×	✓	Low handgrip strength (M: < 28 kg, F: < 18 kg) or Slow 6-m walk: < 1.0 m/s
×	×	✓	✓	Low appendicular skeletal muscle mass measured by Bioelectrical impedance analysis (M: < 7.0 kg/m^2^, F: < 5.7 kg/m^2^)

According to Asian Working Group for Sarcopenia: 2019 Consensus Update on Sarcopenia Diagnosis and Treatment.

Two classification methods were used to discriminate between possible-sarcopenia and robust subjects: ML analysis and a self-developed scoring system. For the ML analysis (details of which are presented in the Supplemental Materials), the features of pulse signals were collected from the 40 indices for each pulse: *C*_*n*_, *CV*_*n*_, *P*_*n*_, and *P*_*n*__*SD* values for *n* = 1–10. The following eight supervised methods were used for the binary classification of the data: support vector machine (SVM), multilayer perceptron (MLP), Gaussian Naïve Bayes (GNB), decision tree (DT), random forest (RF), logistic regression (LR), linear discriminant analysis (LDA), and *k*-nearest neighbor (KNN). Threefold cross-validation was used in the model training process. The confusion matrix was determined, and the accuracy, sensitivity, specificity, and AUC were calculated to evaluate the performance of each classifier.

To further understand the discrimination ability using pulse indices, the self-developed scoring system applied the following steps:

- Selection of the pulse indices that differed either significantly or marginally significantly (0.05 < *p* < 0.1) (according to Fig. 3) between possible-sarcopenia and robust subjects.
- For each selected index, two scores (score1 for the robust bar and score2 for the possible-sarcopenia bar) were assigned in the range from 0 to 10 points according to the definition in the figure below: left, robust < possible sarcopenia; right, robust > possible sarcopenia.
- The score difference for each selected index between score1 and score2 (score1-score2) was calculated.
- For each subject, the average of the score differences for all the selected indices was then calculated to evaluate the similarity of the pulse waveform with a robust characterization (score1-score2 > 0) and a possible-sarcopenia characterization (score1-score2 < 0).

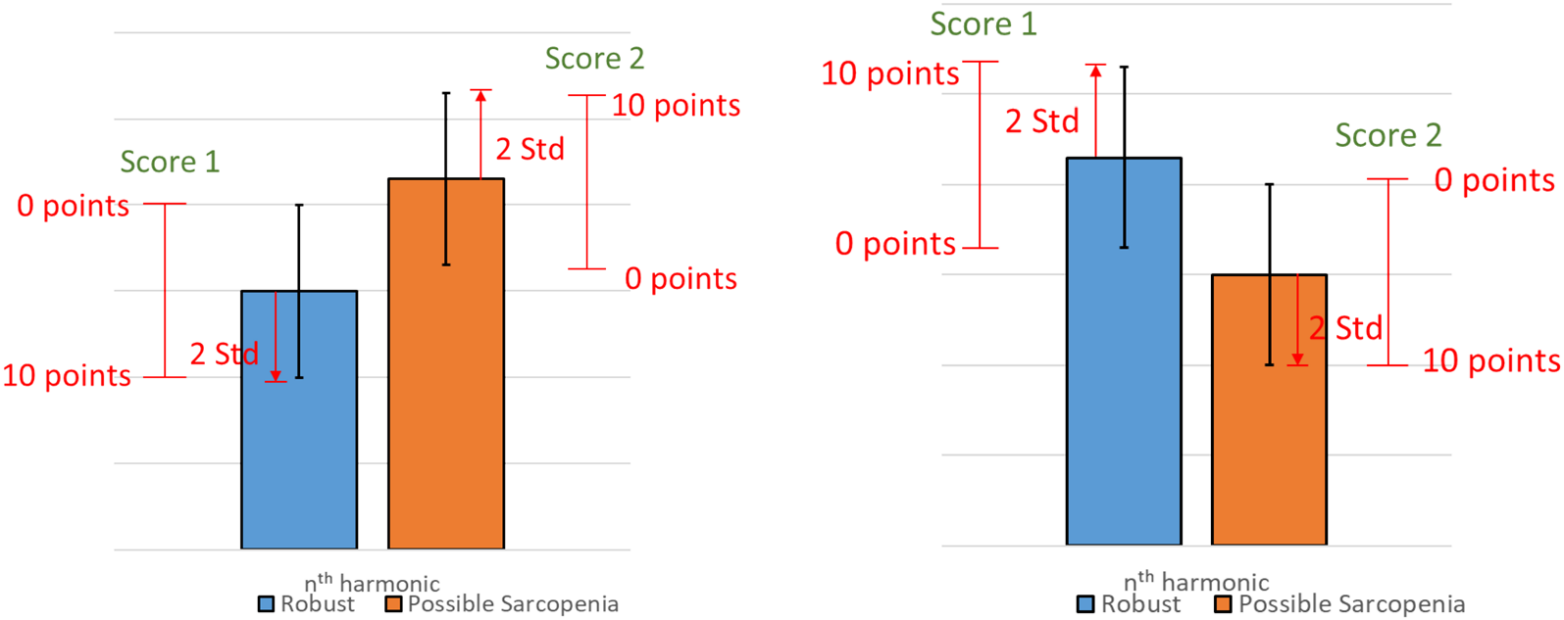


### Informed consent

Informed consent was obtained from all individual participants included in the study.

## Result

**Figure 2:**
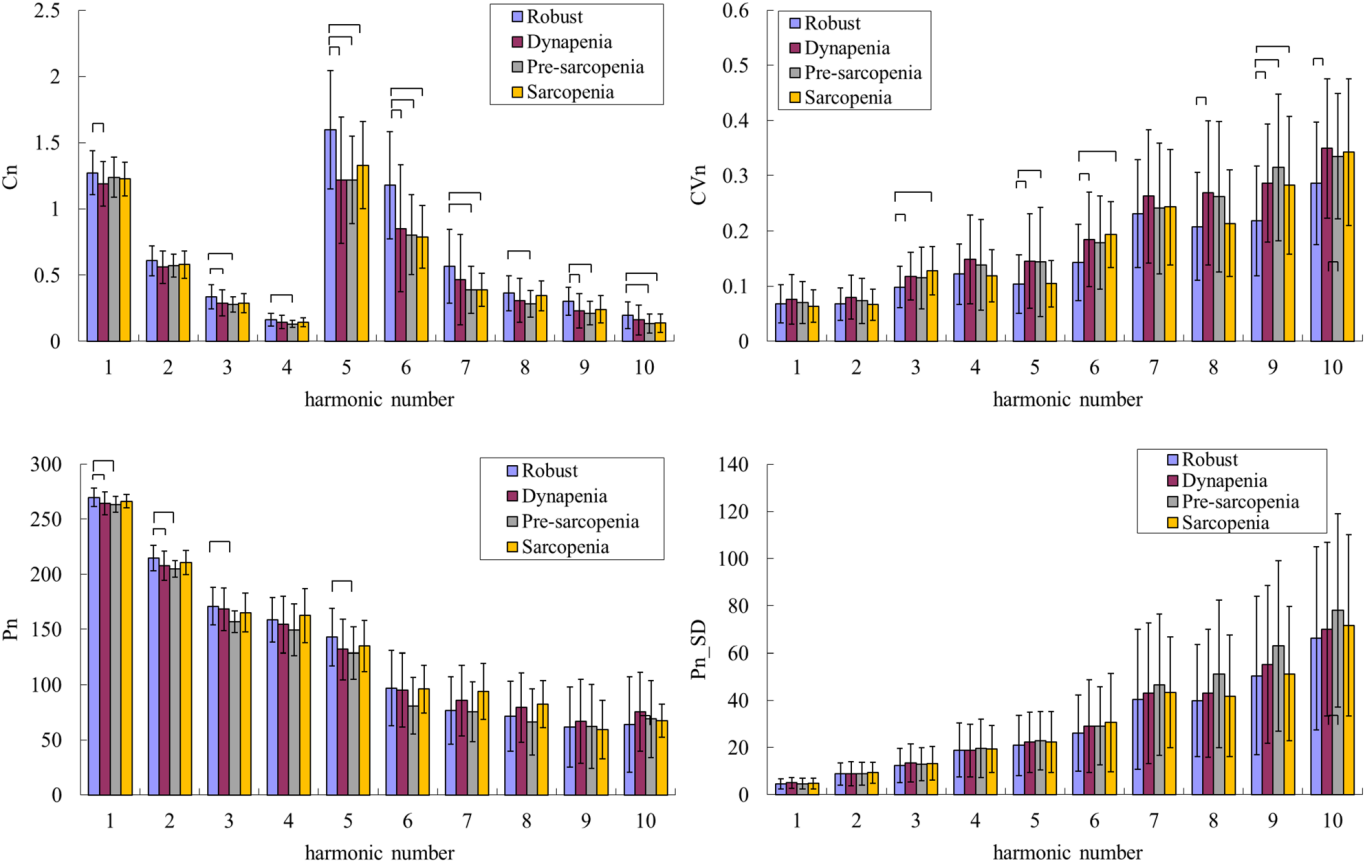
Comparisons of BPW harmonic indices among robust, dynapenia, presarcopenia, and sarcopenia: *C*_*n*_, *CV*_*n*_, *P*_*n*_, and *P*_*n*__*SD*. Data are mean and SD values. *C*_5_–*C*_10_ values have been multiplied by 10 to make the differences clearer. “” indicates *p* < 0.05.

**Figure 3:**
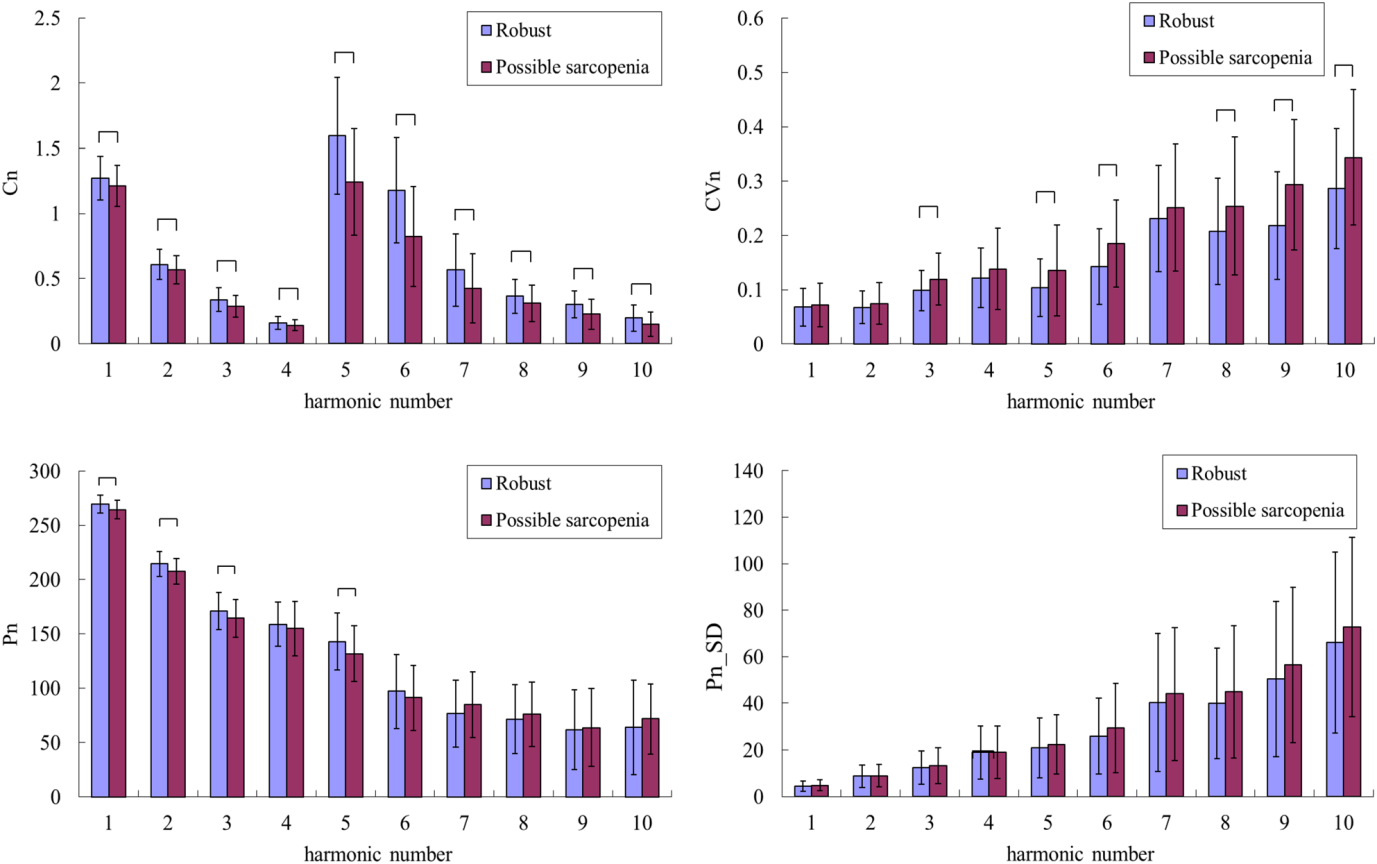
Comparisons of BPW harmonic indices between robust and possible sarcopenia (dynapenia + presarcopenia + sarcopenia): *C*_*n*_, *CV*_*n*_, *P*_*n*_, and *P*_*n*__*SD*. Data are mean and SD values. *C*_5_–*C*_10_ values have been multiplied by 10 to make the differences clearer. “” indicates *p* < 0.05. *C*_*n*_ values were all significantly smaller in possible-sarcopenia subjects than in robust subjects. *CV*_3_–*CV*_10_ were larger in possible-sarcopenia subjects than in robust subjects (significant except for *CV*_4_ and *CV*_7_). *P*_1_–*P*_3_ and *P*_5_ were significantly smaller in possible-sarcopenia subjects than in robust subjects. There were no significant differences in *P*_*n*__*SD*.

The general, clinical, and biochemical characteristics of the participants are listed in Table 1. Figure 2 compares the harmonic indices of the BPW signals among robust, dynapenia, presarcopenia and sarcopenia, and Fig. 3 compares robust and possible-sarcopenia (dynapenia + presarcopenia + sarcopenia) subjects. Figure 3 reveals that among the pulse waveform indices, the *C*_*n*_ values, *P*_1_–*P*_3_, and *P*_5_ were significantly smaller in possible-sarcopenia subjects than in robust subjects. For the pulse variability indices, *CV*_3_–*CV*_10_ were larger in possible-sarcopenia subjects than in robust subjects (all significant except for *CV*_4_ and *CV*_7_). There were no significant differences in *P*_n__*SD*.

**Figure 4:**
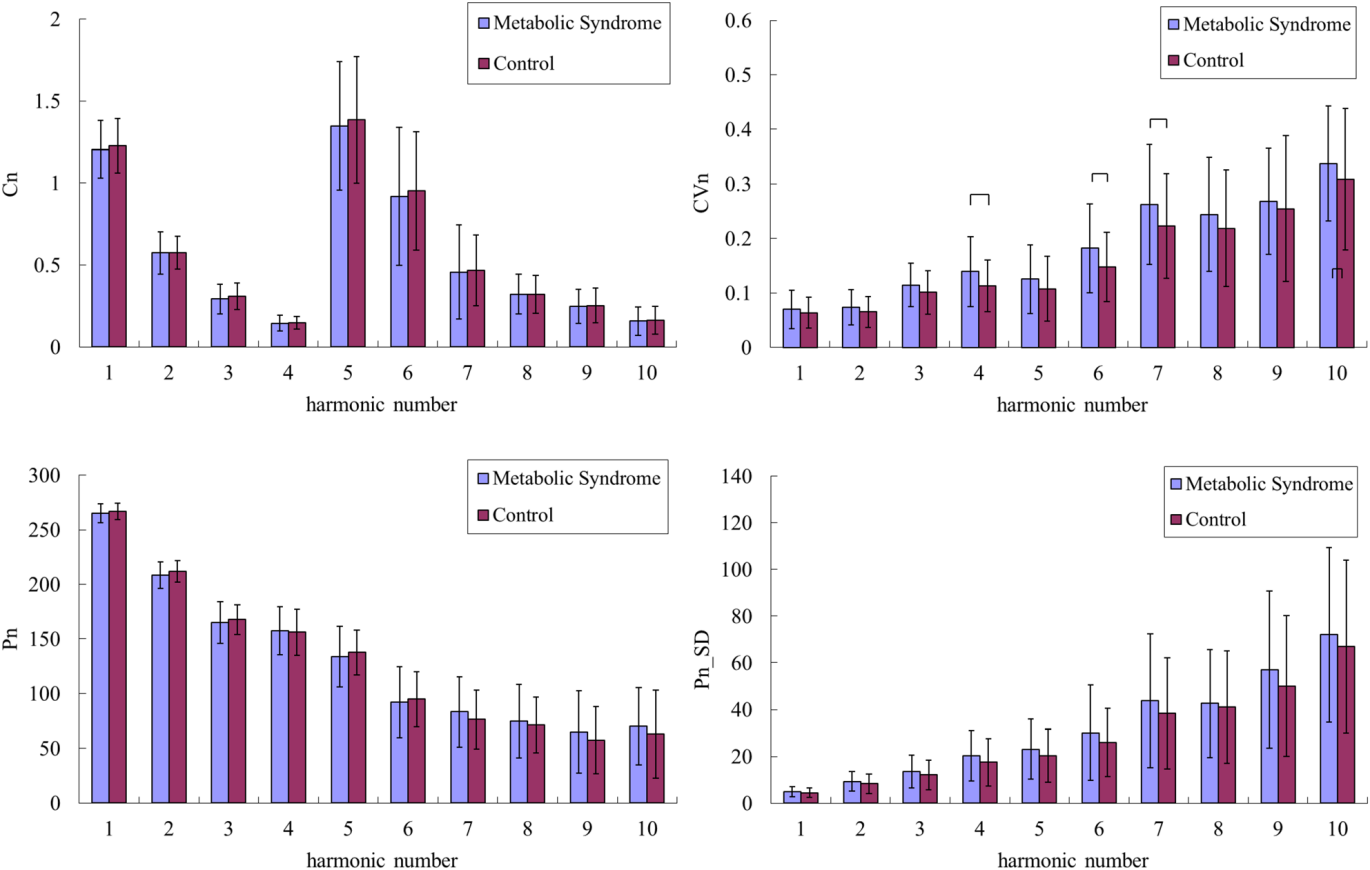
Comparisons of BPW harmonic indices in all subjects between with and without pre-MetS: *C*_*n*_, *CV*_*n*_, *P*_*n*_, and *P*_*n*__*SD*. Data are mean and SD values. *C*_5_–*C*_10_ values have been multiplied by 10 to make the differences clearer. “” indicates *p* < 0.05. Only *CV*_4_, *CV*_6_, and *CV*_7_ differed significantly between with and without pre-MetS.

**Figure 5:**
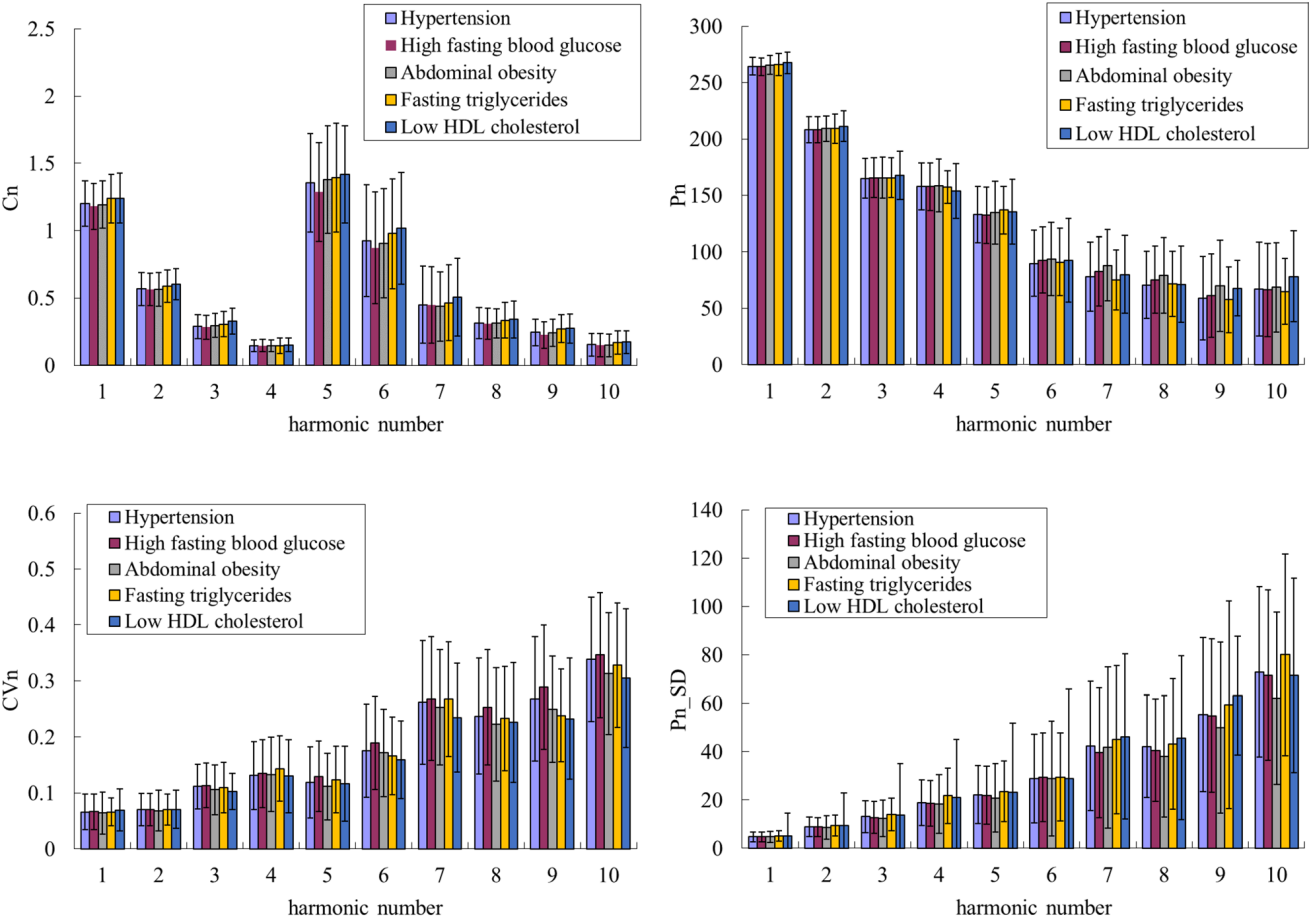
Comparisons of BPW harmonic indices in all subjects with MetS factors. *C*_*n*_, *CV*_*n*_, *P*_*n*_, and *P*_*n*__*SD*. Data are mean and SD values. *C*_5_–*C*_10_ values have been multiplied by 10 to make the differences clearer. There were no any significant differences.

**Figure 6:**
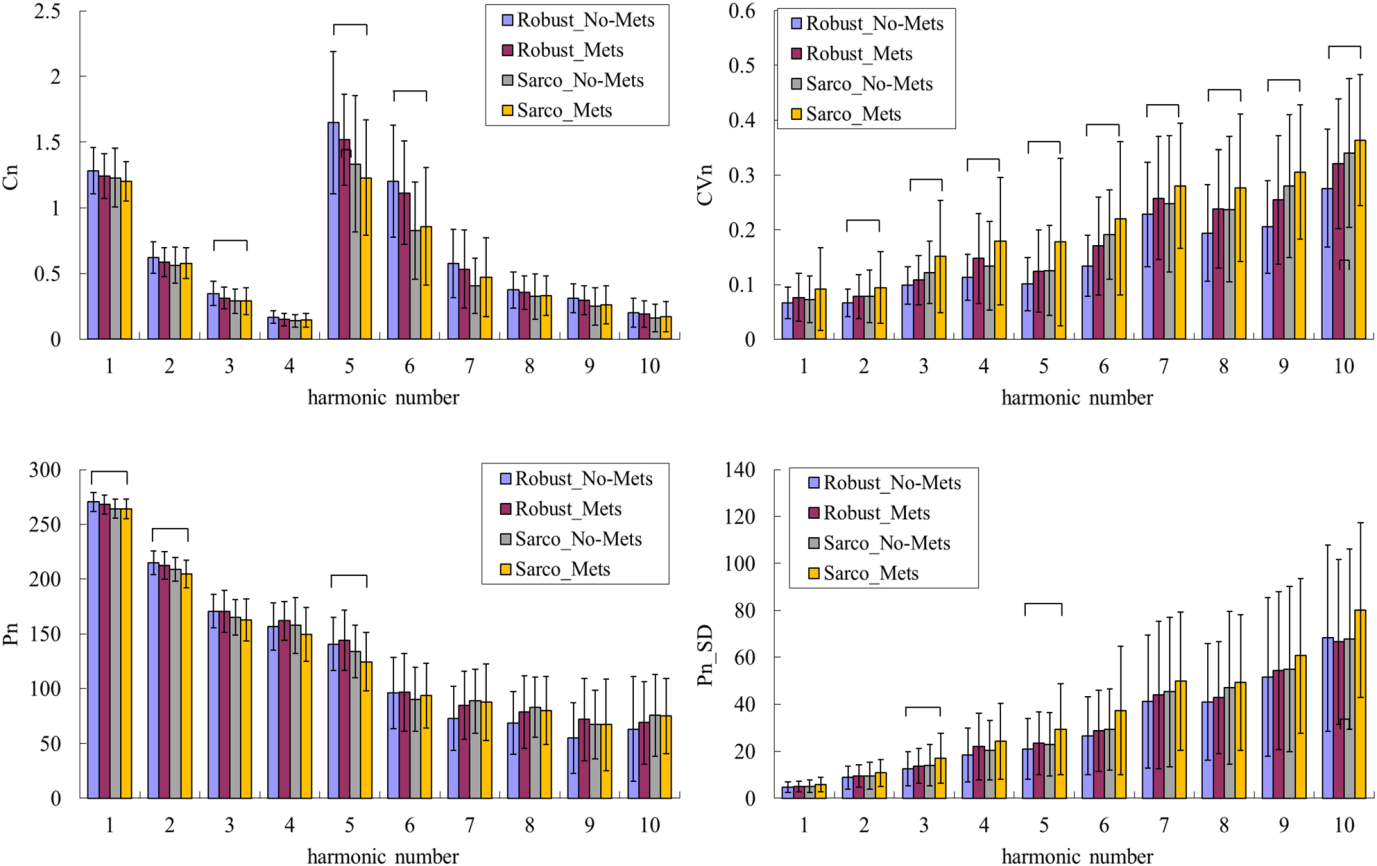
Comparisons of BPW harmonic indices in robust and possible-sarcopenia subjects between with and without pre-MetS: *C*_*n*_, *CV*_*n*_, *P*_*n*_, and *P*_*n*__*SD*. Data are mean and SD values. *C*_5_–*C*_10_ values have been multiplied by 10 to make the differences clearer. “” indicates *p* < 0.05. There were significant differences between robust + no-MetS subjects and possible-sarcopenia + pre-MetS subjects in *C*_5_, *CV*_2_–*CV*_10_, *P*_1_, *P*_2_, *P*_5_, *P*_3__*SD*, and *P*_5__*SD*; many of these indices showed monotonically increasing or decreasing trends in the four subgroups. The differences in the pulse indices relative to the robust + no-MetS subjects were largest for possible-sarcopenia + pre-MetS subjects.

Figure 4 compares the harmonic indices of the BPW signals between with and without pre-MetS. Only *CV*_4_, *CV*_6_, and *CV*_7_ differed significantly between with and without pre-MetS. Figure 5 compares BPW harmonic indices in all subjects with MetS factors. Figure 6 compares the harmonic pulse indices in robust and possible-sarcopenia subjects between with and without pre-MetS. The differences in the pulse indices relative to robust + no-MetS subjects were largest for possible-sarcopenia + pre-MetS subjects. Many of these indices showed monotonically increasing or decreasing trends in the four subgroups. There were significant differences between robust + no-MetS subjects and possible-sarcopenia + pre-MetS subjects in *C*_5_, *CV*_2_–*CV*_10_, *P*_1_, *P*_2_, *P*_5_, *P*_3__*SD*, and *P*_5__*SD*.

**Figure 7:**
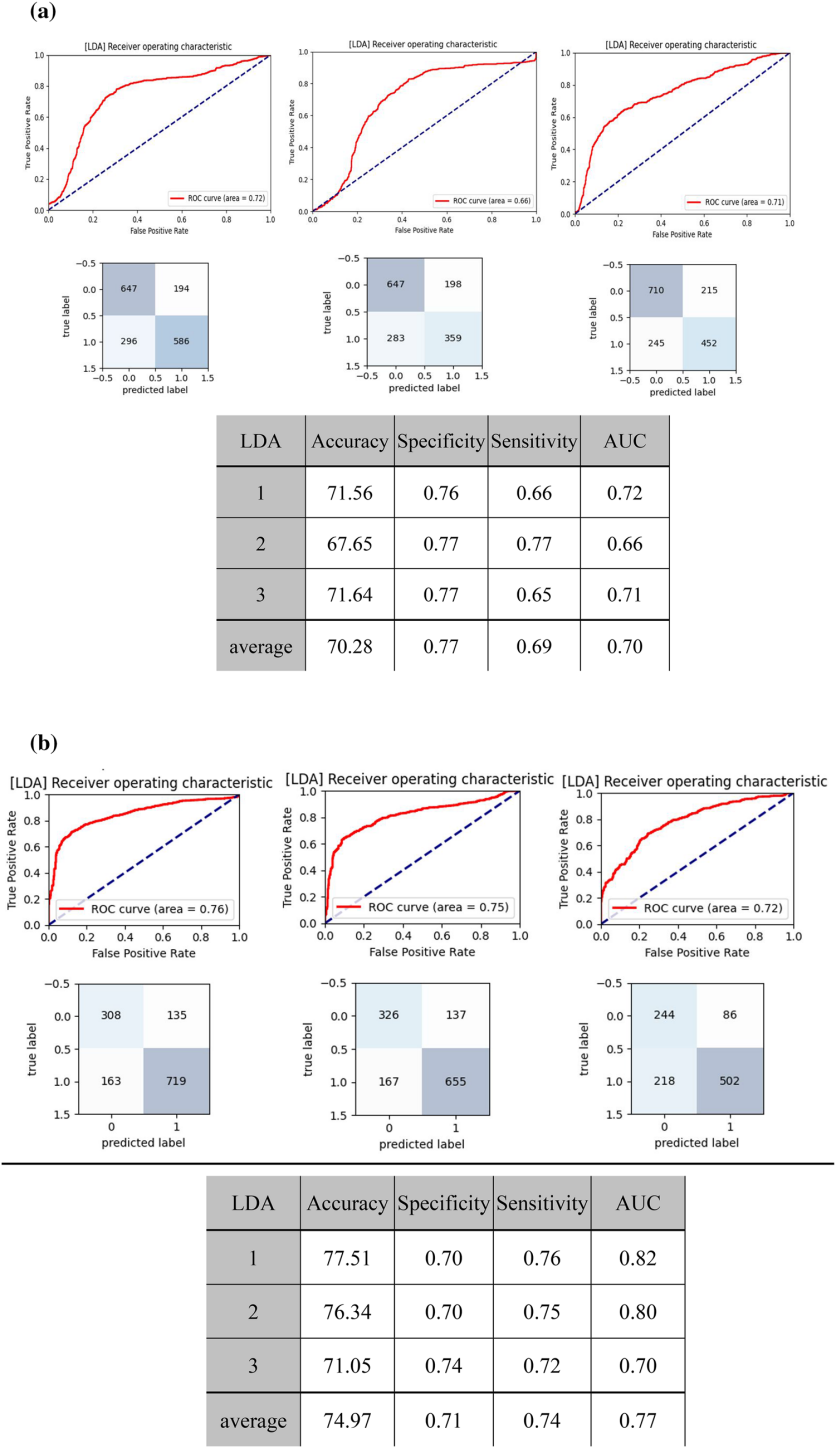
Results of ML analysis (LDA) of BPW indices. (**a**) discriminating between robust and possible sarcopenia. (**b**) discriminating between robust + no-MetS and possible sarcopenia.

Figure 7a and Table 3 present the results of the ML analysis in classifying the subjects into robust and possible sarcopenia. The accuracy and AUC were highest for LDA. The mean accuracy, sensitivity, specificity, and AUC for LDA were 70.28%, 0.69, 0.77, and 0.70, respectively.

**Table 3:** Results of ML analysis (eight methods) of BPW indices for discriminating between robust and possible sarcopenia (dynapenia + presarcopenia + sarcopenia).

Accuracy	SVM	MLP	GNB	DT	RF	LR	LDA	KNN
1	53.40	61.06	49.04	53.92	59.08	58.44	71.56	55.95
2	62.21	64.56	53.46	56.42	64.56	60.73	67.65	59.05
3	57.21	55.55	51.23	56.97	61.71	55.24	71.64	60.11
Average	57.60	60.39	51.25	55.77	61.79	58.14	*70.28	58.37

Sensitivity	SVM	MLP	GNB	DT	RF	LR	LDA	KNN
1	0.34	0.45	0.39	0.4	0.45	0.42	0.66	0.39
2	0.4	0.53	0.34	0.56	0.44	0.43	0.55	0.32
3	0.47	0.61	0.42	0.62	0.65	0.45	0.64	0.48
Average	0.4	0.53	0.38	0.52	0.51	0.43	*0.61	0.39

Specificity	SVM	MLP	GNB	DT	RF	LR	LDA	KNN
1	0.73	0.77	0.59	0.67	0.73	0.75	0.76	0.73
2	0.78	0.73	0.68	0.56	0.8	0.73	0.76	0.79
3	0.64	0.5	0.57	0.52	0.58	0.62	0.76	0.68
Average	0.716	0.66	0.613	0.58	0.7	0.7	*0.76	0.73

AUC	SVM	MLP	GNB	DT	RF	LR	LDA	KNN
1	0.54	0.61	0.49	0.54	0.59	0.59	0.72	0.56
2	0.60	0.63	0.51	0.56	0.62	0.59	0.66	0.56
3	0.56	0.56	0.50	0.58	0.62	0.54	0.71	0.59
Average	0.56	0.60	0.50	0.56	0.61	0.57	*0.70	0.57

The accuracy is in %. Values are for threefold cross-validation. Asterisks indicate the highest average value. Accuracy, sensitivity, specificity and AUC were all highest for LDA.

**Figure 8:**
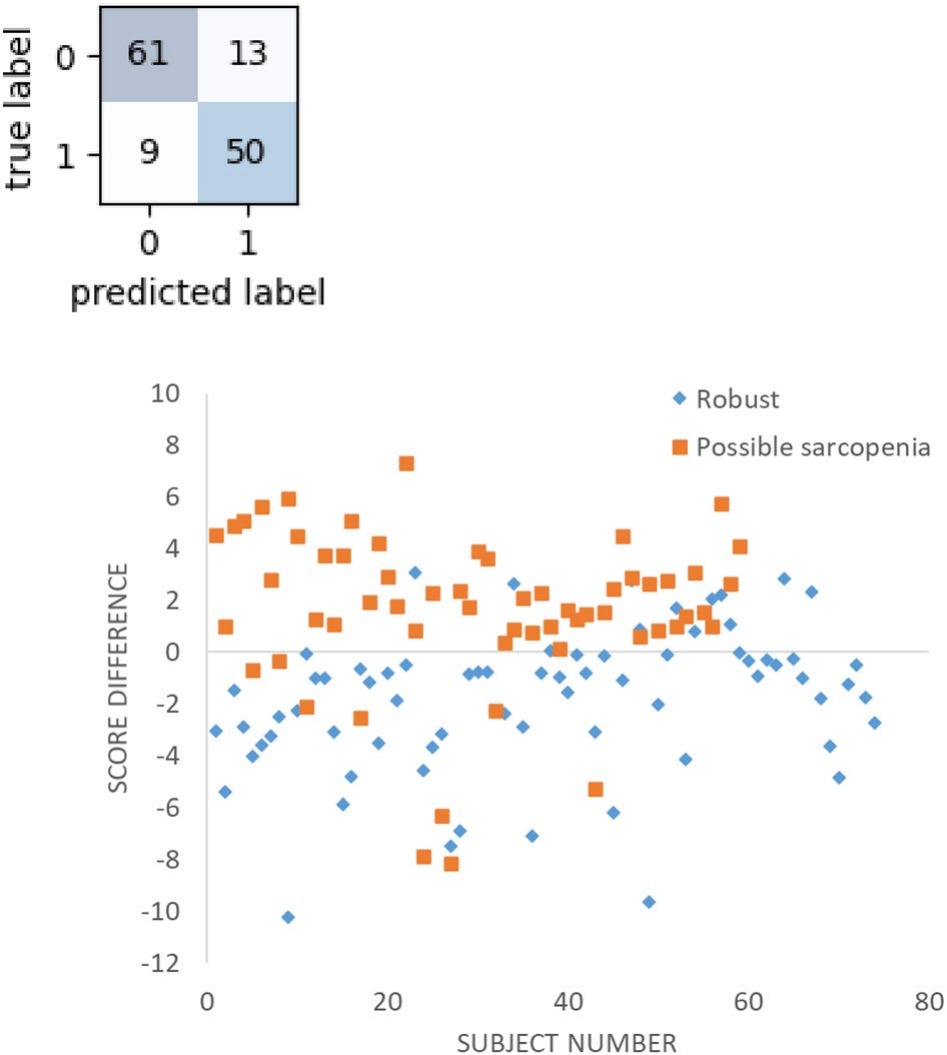
Results of score analysis of BPW indices for discriminating between robust (0) and possible sarcopenia (1). The accuracy, sensitivity, specificity, and AUC were 83.5%, 0.85, 0.82, and 0.83, respectively. Score difference (score1-score2) > 0 indicates robust characterization; score difference < 0 indicates possible-sarcopenia characterization.

Figure 8 presents the confusion matrix for evaluating the performance of using the new scoring system in classifying the subjects into robust and possible-sarcopenia groups. The accuracy, sensitivity, specificity, and AUC for LDA were 83.5%, 0.85, 0.82, and 0.83, respectively. This is better than the performance found when the using LDA.

**Figure 9:**
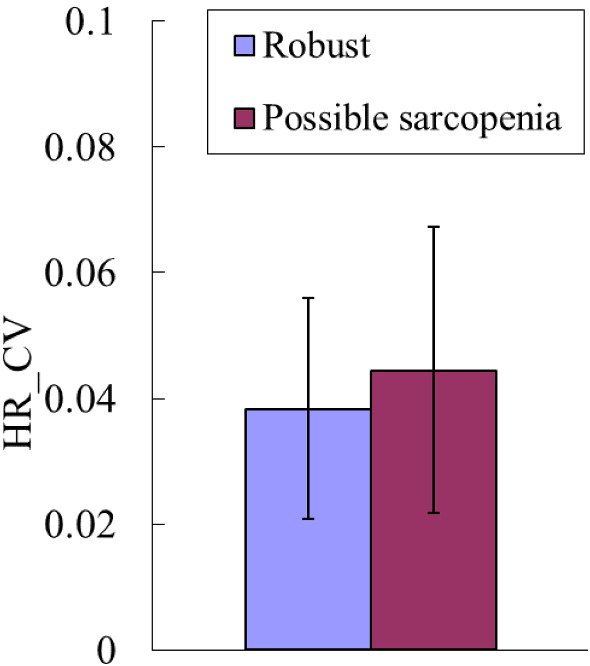
HR_CV did not differ significantly between robust and possible sarcopenia.

Figure 9 shows that HR_CV did not differ significantly between robust and possible sarcopenia. Figure 7b and Table 4 present the results of ML analysis of discrimination between robust + no-MetS vs possible sarcopenia. Among the eight methods, LDA had the highest accuracy and AUC. The mean accuracy, specificity, sensitivity, and AUC for LDA were 74.97%, 0.71, 0.74, and 0.77, respectively.

**Table 4:** Results of ML analysis (eight methods) of BPW indices for discriminating between robust + no-MetS and possible sarcopenia (dynapenia + presarcopenia + sarcopenia).

Accuracy	SVM	MLP	GNB	DT	RF	LR	LDA	KNN
1	62.87	62.64	56.45	58.87	66.72	61.66	77.51	64.23
2	63.42	58.13	50.51	61.17	68.17	55.25	76.34	57.98
3	74.57	68.29	56.10	62.29	70.86	72.29	71.05	55.33
Average	66.95	63.02	54.35	60.77	68.58	63.07	*74.97	59.18

AUC	SVM	MLP	GNB	DT	RF	LR	LDA	KNN
1	0.52	0.56	0.58	0.53	0.61	0.54	0.76	0.61
2	0.56	0.54	0.51	0.59	0.65	0.49	0.75	0.57
3	0.69	0.67	0.62	0.62	0.66	0.67	0.72	0.59
Average	0.59	0.59	0.57	0.58	0.64	0.57	*0.74	0.59

The accuracy is in %. The accuracy and AUC values are listed for the threefold cross validation. Asterisks indicate the highest average value. Both accuracy and AUC were highest for LDA.

## Discussion

This study found that some of the analyzed spectral BPW indices differed significantly between subjects with possible sarcopenia (dynapenia + presarcopenia + sarcopenia) and robust subjects. The differences relative to robust subjects were largest for subjects with combined possible sarcopenia and pre-MetS. The results obtained in the ML analysis (LDA) and from the self-developed scoring system indicated that these differences can be used to aid the discrimination of possible sarcopenia.

### Comparison in pulse indices

It has been suggested that sarcopenia is associated with vascular lesions and poor microcirculation^10^. For example, the capillary density in skeletal muscle has been found to be low in sedentary older adults with sarcopenia^6^. Significant positive correlations were found between skin perfusion pressure and appendicular muscle mass^10^. PAD can result in decreased blood flow to the lower extremities, with the underlying pathophysiology characterized by metabolic and structural myopathic changes in skeletal muscles^11^. Decreases in muscle mass and strength were associated with endothelial dysfunction. Risk factors for atherosclerosis (BP and cholesterol) were noted to be significantly higher in sarcopenia; decreased skeletal muscle mass is an independent risk factor for arteriosclerosis vascular disease^8^.

Figure 3 indicates that the amplitude proportions differed significantly between possible-sarcopenia and robust subjects, with all *C*_*n*_ values being significantly smaller in possible sarcopenia. This illustrates that the BPW can be changed by the effects exerted by sarcopenia on the vascular elastic properties, and hence partly account for the mechanisms that allow *C*_*n*_ indices to be utilized in the discrimination of sarcopenia.

It is possible that the decreased *C*_*n*_ values are related to decreased transmission efficiency of frequency components within the BPW induced by sarcopenia. The factors underlying the pathogenesis of sarcopenia include low physical activity and increased oxidative stress and inflammatory factors, which have been noted to induce changes in the arterial stiffness and promote atherosclerosis^2^. Inflammation and oxidative stress have been suggested to be the two most important factors underlying the common pathogenesis of sarcopenia and atherosclerosis^8^. Long-term systemic chronic inflammation is involved in CVD and sarcopenia in the elderly^8^. For example, inflammatory factors such as tumor necrosis factor and interleukin-6 are essential catabolic factors that can stimulate protein catabolism, inhibit muscle synthesis, and promote muscle atrophy. Interleukin-6 is negatively associated with muscle mass and strength^8^. Oxidative stress is a common mechanism in many age-related diseases, and is crucial to the pathogenesis of both CVDs (e.g., hypertension and atherosclerosis) and sarcopenia^8^. Reactive oxygen species can affect muscle protein synthesis, enhance the hydrolysis of muscle protein, and lead to sarcopenia. Plasminogen activator inhibitor-1 can induce fibrinolysis abnormalities, which may lead to CVD via dysregulation of vascular clotting, endothelial dysfunction, and metabolic abnormalities^1^.

Figure 3 also reveals significant differences in *P*_*n*_ values between possible-sarcopenia and robust subjects. The amplitude and phase angle are two main indices that can be calculated using spectral analysis from a time-domain waveform. Since they are linearly independent of each other, *P*_*n*_ values can not only provide another set of indices to describe the BPW waveform in more detail, but can also be used to increase the number of features when performing ML analysis. The physical meaning of *P*_*n*_ is related to the time delay to the onset point and it can be correlated with the wave propagation velocity of each frequency component within the BPW, and hence it could be useful as another index for evaluating changes in vascular elastic properties.

Figure 3 shows that *P*_*n*_ values were smaller for lower-frequency components (*P*_1_–*P*_6_; significantly in *P*_1_, *P*_2_, *P*_3_, and *P*_5_) in possible-sarcopenia subjects than in robust subjects, which leads to relatively larger phase-angle values for the higher-frequency components. The main (lower-frequency) frequency components constituting a larger proportion of the total BPW power could be more related to the pulse transmission in the main artery, and the higher-frequency components could be more related to the pulse transmission in the peripheral arteries. It is possible that the higher stiffness of peripheral arteries in possible sarcopenia make the higher-frequency components within the BPW transmit along the artery more rapidly, hence leading to their larger *P*_*n*_ values.

### Comparisons of pulse variability indices

Cardiovascular variability analysis has been widely applied to monitor the regulatory activities of the cardiovascular system induced by external stimulation or the physiological effects of diseases. For example, analyses of HR and BP variability have been used to investigate changes related to diseases and aging^23–25^. Similarly, variability of frequency-domain BPW indices (*CV*_*n*_ and *P*_*n*__*SD*) can be used to monitor the regulatory efforts induced by changes in the blood flow or vascular stiffness condition^21,22^.

Figure 3 indicates that *CV*_*n*_ values were larger for higher-frequency components (*CV*_3_–*CV*_10_; significant except for *CV*_4_ and *CV*_7_) in possible-sarcopenia subjects than in robust subjects. This could be at least partly attributed to the following three mechanisms: (1) sarcopenia can be accompanied with impairment of the blood supply to the peripheral vascular beds, which could increase the regulatory efforts needed for peripheral vessels such as when restoring normal blood perfusion, and hence increase the instability of pulse indices; (2) sarcopenia-induced effects such as dysregulation of vascular clotting, endothelial dysfunction, and metabolic abnormalities^1^ can increase the instability of pulse indices; and (3) the worse blood supply to peripheral vessels makes it more difficult to radially distend the vessel and hence decrease the pulse pressure, which can reduce the signal-to-noise ratio of the pulse signal and thereby lead to instability of pulse indices.

The findings in Fig. 9 indicate that HR_CV did not differ between possible-sarcopenia and robust subjects. HR_CV was calculated from the R–R intervals of the ECG signals; it is correlated with the regulatory activities for the heartbeat, and can have different physiological meanings from the present pulse variability indices since the latter might be more strongly correlated with the peripheral circulation. The present findings illustrate that even in the absence of a significant difference in the HR variability index, the pulse variability indices can still aid the discrimination of possible sarcopenia. The possible underlying mechanisms could be at least partly related to the effects of sarcopenia on the peripheral circulation, such as increased vascular stiffness and poor microcirculation.

### Effects of pre-MetS

Figure 4 indicates that there were only a few significant differences between subjects with and without pre-MetS. The comparison of the robust and possible-sarcopenia subjects between those with and without pre-MetS shown in Fig. 6 indicates that the differences were more prominent—characterized by more indices with significant differences and larger differences in average values—for the pulse indices in subjects with combined pre-MetS and possible sarcopenia than in the robust subjects.

Similar to sarcopenia, MetS could be associated with dysfunction and impairments of both the macro- and microcirculation^18,26^. Morphological and functional disorders of large vessels, endothelium-dependent dysfunction of the microcirculation, lower density of functional capillaries, and higher skin microcirculation resistance have been noted in MetS^26^. The decreased blood flow to the extremities can affect muscle mass and strength, which correspond to the definition of sarcopenia. It is possible that combined MetS and sarcopenia exerts larger effects on the peripheral circulation (perhaps including vascular and microcirculatory effects), hence resulting in more-prominent differences in pulse indices. Effects of PAD (which can affect the peripheral circulation) can accompany sarcopenia in decreased mobility and impaired PAD-specific mobility^11^. MetS is suggested to be associated with an increased risk of CVDs, and the present findings have demonstrated that pulse analysis can be used to monitor changes in vascular condition in sarcopenia combined with pre-MetS.

### Discrimination by ML analysis and the new scoring system

It is suggested that developing cost-effective risk prediction models for sarcopenia in clinical settings is difficult^9^. ML analysis has been applied to automate CT segmentation to improve evaluations of muscle, bone, and adipose tissues^5^. ML analysis using medical records of human subjects as features (e.g., BMI, RBC count, WBC count, BUN, and primary diastolic BP) has achieved an AUC of around 0.8^9^, which supports the possibility of using ML analysis in sarcopenia discrimination. The present study applied ML analysis to assess its utility in discriminating between possible-sarcopenia and robust subjects. Figure 7a and Table 3 reveal that LDA had the highest AUC (0.70, which represents acceptable discrimination) among the eight ML analysis methods used in this study.

40 pulse indices were used as features in the present study. LDA is a dimensionality reduction algorithm, which can effectively save the operation cost in the case of large number of features. It is possible that the LDA transforms the features from high dimension to low dimension through mapping, makes the data differences between different categories greater, and reduces the distance between the data in the same categories, so that the data points after dimensionality reduction are easier to distinguish.

We also developed a scoring system for discriminating between possible-sarcopenia and robust subjects. Figure 8 shows that this analysis method achieved an AUC of 0.83, which achieved excellent discrimination, and was better than the result obtained in the ML analysis. This can be compared with the result obtained in a previous study that used medical records as features^9^. We have demonstrated a time-saving and easy-to-use measurement and analysis method for sarcopenia discrimination.

Since the ultimate aim of this study was to improve the early screening of sarcopenia, our discrimination analysis was focused on possible sarcopenia (and also the effects of pre-MetS), which is a greater challenge than sarcopenia discrimination. An important limitation of the study was the relatively small number of sarcopenia subjects, since they were recruited during geriatric health checkups that mostly involved nonpatients. The acquisition of data on more subjects might result in the two methods having similar discrimination performance, making it possible that future ML analyses will achieve higher AUC values in discriminating sarcopenia.

ML analysis was performed to discriminate between robust + no-MetS vs possible sarcopenia in Fig. 7b and Table 4. LDA had an AUC of 0.77, which is close to an excellent discrimination performance. This could be partly attributed to the effects on the vascular elastic properties, which can be induced by the pre-MetS and the possible sarcopenia. In practical application, the factor of pre-Mets can be known prior to the pulse measurement; therefore excluding the pre-Mets from the robust can help to further improve the discrimination performance and at the same time do not affect the application convenience of the of the present pulse measurement.

## Conclusion

The findings of this study and the related conclusions to be drawn can be summarized as follows:

- Significant differences were found in several spectral indices of the BPW between possible-sarcopenia subjects (combining dynapenia, presarcopenia, and sarcopenia) and robust subjects.
- Threefold cross-validation results indicated excellent discrimination performance (AUC = 0.70 when using LDA, and AUC = 0.83 when using our scoring system).
- The differences relative to robust subjects were largest for subjects with combined possible sarcopenia and pre-MetS. When excluding the pre-Mets subjects from the Robust, LDA can achieve better performance with AUC = 0.77, which is close to excellent discrimination performance.
- The present noninvasive and easy-to-use measurement and analysis method (comprising ML analysis and the self-developed scoring system) for detecting sarcopenia-induced changes in the arterial pulse transmission condition could aid the discrimination of possible sarcopenia. The possible effects of other factors affecting the elastic properties of blood vessels should also be considered, which is an important work for our future research.

## Supplementary Information

Supplementary Information.

## Data Availability

The datasets generated during and/or analysed during the current study are available from the corresponding author on reasonable request.

## References

[1] Rubio-Ruiz ME, Guarner-Lans V, Pérez-Torres I, Soto ME (2019). Mechanisms underlying metabolic syndrome-related sarcopenia and possible therapeutic measures. Int. J. Mol. Sci..

[2] Rong YD, Bian AL, Hu HY, Ma Y, Zhou XZ (2020). A cross-sectional study of the relationships between different components of sarcopenia and brachial ankle pulse wave velocity in community-dwelling elderly. BMC Geriatr..

[3] Kwak JY, Hwang H, Kim SK, Choi JY, Lee SM, Bang H, Kwon ES, Lee KP, Chung SG, Kwon KS (2018). Prediction of sarcopenia using a combination of multiple serum biomarkers. Sci. Rep..

[4] Beaudart C, Rizzoli R, Bruyère O, Reginster JY, Biver E (2014). Sarcopenia: Burden and challenges for public health. Arch. Public Health.

[5] Lenchik L, Boutin RD (2018). Sarcopenia: Beyond muscle atrophy and into the new frontiers of opportunistic imaging, precision medicine, and machine learning. Semin. Musculoskelet. Radiol..

[6] Addison O, Prior SJ, Kundi R, Serra MC, Katzel LI, Gardner AW, Ryan AS (2018). Sarcopenia in peripheral arterial disease: Prevalence and effect on functional status. Arch. Phys. Med. Rehabil..

[7] Cruz-Jentoft AJ, Bahat G, Bauer J, Boirie Y, Bruyère O, Cederholm T, Cooper C, Landi F, Rolland Y, Sayer AA, Schneider SM, Sieber CC, Topinkova E, Vandewoude M, Visser M, Zamboni M (2019). Writing group for the European working group on sarcopenia in older people 2 (EWGSOP2), and the extended group for EWGSOP2. Sarcopenia: Revised European consensus on definition and diagnosis. Age Ageing.

[8] He N, Zhang Y, Zhang L, Zhang S, Ye H (2021). Relationship between sarcopenia and cardiovascular diseases in the elderly: An overview. Front. Cardiovasc. Med..

[9] Kang YJ, Yoo JI, Ha YC (2019). Sarcopenia feature selection and risk prediction using machine learning: A cross-sectional study. Med. (Baltimore).

[10] Jung CH, Cho YY, Choi D, Kim BY, Kim CH, Mok JO (2020). Relationship of sarcopenia with microcirculation measured by skin perfusion pressure in patients with type 2 diabetes. Endocrinol. Metab. (Seoul).

[11] Pizzimenti M, Meyer A, Charles AL, Giannini M, Chakfé N, Lejay A, Geny B (2020). Sarcopenia and peripheral arterial disease: A systematic review. J. Cachexia Sarcopenia Muscle.

[12] O'Rourke MF, Adji A, Safar ME (2018). Structure and function of systemic arteries: Reflections on the arterial pulse. Am. J. Hypertens..

[13] Johnson JE, Shay O, Kim C, Liao C (2019). Wearable millimeter-wave device for contactless measurement of arterial pulses. IEEE Trans. Biomed. Circuits Syst..

[14] Husmann M, Jacomella V, Thalhammer C, Amann-Vesti BR (2015). Markers of arterial stiffness in peripheral arterial disease. Vasa.

[15] Mackenzie IS, Wilkinson IB, Cockcroft JR (2002). Assessment of arterial stiffness in clinical practice. QJM.

[16] Davies JI, Struthers AD (2005). Beyond blood pressure: Pulse wave analysis–a better way of assessing cardiovascular risk?. Future Cardiol..

[17] Bor-Seng-Shu E, Kita WS, Figueiredo EG, Paiva WS, Fonoff ET, Teixeira MJ, Panerai RB (2012). Cerebral hemodynamics: Concepts of clinical importance. Arq. Neuropsiquiatr..

[18] Chang YW, Hsiu H, Yang SH, Fang WH, Tsai HC (2016). Characteristics of beat-to-beat photoplethysmography waveform indexes in subjects with metabolic syndrome. Microvasc. Res..

[19] Lin SK, Hsiu H, Chen HS, Yang CJ (2021). Classification of patients with Alzheimer’s disease using the arterial pulse spectrum and a multilayer-perceptron analysis. Sci. Rep..

[20] Lin FL, Hsiu H, Chiu HS, Chen CT, Hsu CH (2020). Characteristics of pulse-waveform and laser-Doppler indices in frozen-shoulder patients. Biomed. Signal Process Control.

[21] Hsiu H, Liu JC, Yang CJ, Chen HS, Wu MS, Hao WR, Lee KY, Hu CJ, Wang YH, Fang YA (2022). Discrimination of vascular aging using the arterial pulse spectrum and machine-learning analysis. Microvasc. Res..

[22] Hsiu H, Lin SK, Weng WL, Hung CM, Chang CK, Lee CC, Chen CT (2022). Discrimination of the cognitive function of community subjects using the arterial pulse spectrum and machine-learning analysis. Sensors (Basel).

[23] Stergiou GS, Ntineri A, Kollias A, Ohkubo T, Imai Y, Parati G (2014). Blood pressure variability assessed by home measurements: A systematic review. Hypertens. Res..

[24] Task Force of the European Society of Cardiology and the North American Society of Pacing and Electrophysiology (1996). Heart rate variability. Standards of measurement, physiological interpretation, and clinical use. Eur. Heart J..

[25] Freitas VP, Passos RDS, Oliveira AA, Ribeiro ÍJS, Freire IV, Schettino L, Teles MF, Casotti CA, Pereira R (2018). Sarcopenia is associated to an impaired autonomic heart rate modulation in community-dwelling old adults. Arch. Gerontol. Geriatr..

[26] Czernichow S, Greenfield JR, Galan P, Jellouli F, Safar ME, Blacher J, Hercberg S, Levy BI (2010). Macrovascular and microvascular dysfunction in the metabolic syndrome. Hypertens. Res..

