# Supplementary material for: Discrimination between possible sarcopenia and metabolic syndrome using the arterial pulse spectrum and machine-learning analysis
Source: Sci Rep. 2022 Dec 12;12:21452. doi: 10.1038/s41598-022-26074-5 (PMC9744729; doi:10.1038/s41598-022-26074-5)
Supplement: Supplementary file 1 — Supplementary Information. [file 41598_2022_26074_MOESM1_ESM.doc]

Title: Discrimination between possible sarcopenia and metabolic syndrome using the arterial pulse spectrum and machine-learning analysis

Short title: ML pulse classification of sarcopenia

Authors: Li-Wei Wu^1, 2^, Te OuYoung^1, 2^, Yu-Chih Chiu^3^, Ho-Feng Hsieh^3^, Hsin Hsiu^3, 4^

1. Division of Family Medicine, Department of Family and Community Medicine, Tri-Service General Hospital; School of Medicine, National Defense Medical Center, Taipei, Taiwan.

2. Health Management Center, Department of Family and Community Medicine, Tri-Service General Hospital, National Defense Medical Center, Taipei, Taiwan.

3. Graduate Institute of Biomedical Engineering, National Taiwan University of Science and Technology, Taipei, Taiwan.

4. Biomedical Engineering Research Center, National Defense Medical Center, Taipei, Taiwan.

To whom correspondence should be addressed: Hsin Hsiu, PhD.

Hsin Hsiu: No.43, Section 4, Keelung Road, Graduate Institute of Biomedical Engineering, National Taiwan University of Science and Technology, Taipei 10607, Taiwan.

**1. Measurements**

Before the measurements, the subjects were relaxed and rested for at least 10 min. The environmental temperature was within 23-25 °C during the entire measuring period. All subjects gave their informed consent before experiments commenced, were asked to not take any medication for 3 days before experiments, and did not consume food at least 1 h before each experiment. All subjects were non-smokers, and did not take coffee or drinks containing alcohol at least 1 day before experiments

For each experiment, the subjects were sitting on a chair, and 1-minute ECG and BPW signals were measured noninvasively. ECG signals were measured by surface electrodes, and acquired by a preamplifier (lead II, RA-LL; 6600-series, Gould, USA). The BPW signal was acquired by a pressure transducer (KFG-2-120-D1-11, Kyowa) held onto the skin surface above the radial artery 2 cm from the left wrist. The signals were connected to a self-made current-to-voltage converter circuit, and then connected to an analog-to-digital converter card (PCI- 9111DG, Adlink Technology, Taiwan) operating at a sampling rate of 1024 Hz. Before the measurement, the heart rate (HR), brachial systolic BP and diastolic BP were measured by using a sphygmomanometer (MG150f, Rossmax).

One thermistor was attached around the wrist to monitor the skin-surface temperature. The resistance of the thermistor was transformed into voltages (by a custom-made circuit) that were also sampled every minute by the analog-to-digital converter card. The acceptable range for the temperature stability during the baseline period was a temperature variation of less than 1.0 °C.

**2. Analysis**

The present analysis procedure included signal processing and information processing:

- signal processing

Frequency-domain analysis was applied to derive the 40 harmonic indices from the measured BPW signal (*n*=1-10): amplitude proportion (*C_n_*), coefficient of variation of *C_n_* (*CV_n_*), phase angle (*P_n_*), and standard deviation of *P_n_* (*Pn*_*SD*).

Each individual pulse (between foot points) can be represented by the following finite series. The pulses were excluded if the values between the two foot points were larger than 20% of the pulse amplitude.

$x(t)=\frac{A_{0}}{2}+\left\{\sum_{n=1}^{k/2} A_{n}\cos n\omega t_{s}+\sum_{n=1}^{k/2} B_{n}\cos n\omega t_{s}\right\}$

The Fourier coefficients (*A_n_* and *B_n_*) of the pulse can be calculated as

$\begin{matrix} A_{n}=\frac{2}{k}\sum_{s=0}^{k} x_{s}\cos n\omega t_{s\text{ }}(for\text{ }n=0,1,...,\frac{k}{2}) \\ B_{n}=\frac{2}{k}\sum_{s=0}^{k} x_{s}\sin n\omega t_{s\text{ }}(for\text{ }n=0,1,...,\frac{k}{2}) \end{matrix}$

where $\omega$ is the angular frequency and $t_{s}$ is the sampling time interval.

The amplitude (*Amp_n_*) and phase angle (*P_n_*) of each harmonic of the pulse harmonic spectrum can then be calculated as $Amp_{n}=\sqrt{A_{n}^{2}+B_{n}^{2}}$ and $P_{n}=\arctan(B_{n}/A_{n})$. The amplitude proportions (*C_n_* values) for each pulse were calculated as *Amp_n_* / *Amp*_0_ × 100%, for *n* = 1–10. *CV*_n_ was then calculated as the coefficient of variations of *C_n_*, and *P*n_*SD* was calculated as the standard deviation of *P*_n_.

Signal processing was performed with MATLAB (MathWorks). The differences in the fundamental physiological parameters were tested with one-way ANOVA. The Tukey-HSD method was used for post-hoc analysis. The differences in pulse indices were tested with two-tailed t-test and were considered significant when *p*<0.05; all *p*-values were two-sided hypotheses.

- information processing

For information processing, the features of pulse signals were collected from the results of the signal-processing stage described above, to yield 40 indices for each pulse: *C_n_*, *CV_n_*, *P_n_*, and *Pn*_*SD* values for *n* = 1–10. Each feature was scaled by *Z*-score normalization to eliminate the effects of the variations in the ranges of different indices. Python (version 3.7) was used as the analysis tool in the information processing; eight machine-learning methods were used to classify the data (details of model parameters are listed in the following table).

Threefold cross validation was used in the model training stage. The proposed classification model was evaluated by calculating the accuracy, AUC (area under the receiver operating characteristics curve), sensitivity and specificity.

Parameters of the machine-learning models.

| machine-learning methods | model parameters |
| --- | --- |
| SVM  (support vector machine) | C=1; kernel: rbf; gamma: auto; tol= 0.0001; max_iter=-1; class_weight: none |
| MLP  (multilayer perception) | hidden_layer_sizes=100; solver: adam; alpha=0.0001; batch_size: auto; max_iter=200; learning_rate_int=0.001 |
| GNB  (Gaussian Naive Bayes) | Priors: none |
| DT  (decision tree) | Criterion: gini; Splitter: best; max_depth: none; min_samples_split=2; min_samples_leaf=1; min_weight_fraction_leaf=0; max_features: none; max_leaf_nodes: none; min_impurity_split=0.0 |
| RF  (random forest) | n_estimators=100; criterion: gini; max_depth: none; min_samples_split=2; min_samples_leaf=1; min_weight_fraction_leaf=0; max_features: none; max_leaf_nodes: none |
| LR  (logistic regression) | Penalty: l2; Solver: lbfgs; multi_class: auto; class_weight: none |
| LDA  (linear discriminant analysis) | Solver: svd; Shrinkage: none; Priors: none |
| KNN  (K-nearest neighbor classification) | n_neighbors=5; weights: uniform; algorithm: auto; n_jobs: none; p: none |
